# The Influence of Mothers’ Lifestyle and Health Behavior on Their Children: An Exploration for Oral Health

**DOI:** 10.5812/ircmj.16051

**Published:** 2014-02-05

**Authors:** Keramat Nourijelyani, Mir Saeed Yekaninejad, Mohammad Reza Eshraghian, Kazem Mohammad, Abbas Rahimi Foroushani, Amir Pakpour

**Affiliations:** 1 Department of Epidemiology and Biostatistics, Tehran University of Medical Sciences, Tehran, IR Iran; 2 Social Determinants of Health Research Center, Qazvin University of Medical Sciences, Qazvin, IR Iran; 3 Department of Public Health, Qazvin University of Medical Sciences, Qazvin, IR Iran

**Keywords:** Oral Health, Lifestyle, Mothers, Health Behavior

## Abstract

**Background:**

Parents and teachers involvement reinforce health promotion programs for children's health.

**Objectives:**

The purpose of this study was to evaluate mothers’ lifestyle behavior and its association with children's oral health.

**Materials and Methods:**

The study was a cross sectional study on 383 children and their mothers who were selected from 6 primary schools in Tehran, Iran. Mothers and children who participated in this study were asked to complete a questionnaire containing demographic questions, knowledge of oral health, attitude towards the oral health behavior, and oral health behaviors. Furthermore, the Simplified Oral Hygiene Index (OHI-S) and Community Periodontal Index (CPI) were assessed by two calibrated dentists. Data were analyzed with multilevel mixed model analyses.

**Results:**

The average age of the children and their mothers were 11.6 and 38.4 years, respectively. Mothers’ higher knowledge, higher educational status, positive attitude, higher frequent oral health behaviors, lower DMFT and lower CPI were all associated significantly with children’s higher oral health status.

**Conclusions:**

The results suggest that to improve children’s oral health, educational interventions should focus on both children and mothers to obtain a more promising outcome.

## 1. Background

Having healthy teeth is not only related to the oral health but also is associated with the general health and quality of life (1). Oral diseases are major public health problem around the world (2). The prevalence of dental caries has increased in developing countries significantly with most adults and about 60 % of schoolchildren suffering from dental carries (2, 3). A study from Iran demonstrated that the mean values of dmft for 9-year-old boys and girls were 4.2 and 3.4, respectively (4). Also, a recent study in Iran revealed that the mean dmft values are 3.6 and 5.0 for 6 and 9 year old children, respectively (5). These figures are considerably higher than the mean DMFT worldwide (1.63) and even are higher than the Eastern Mediterranean region value (1.67) (6). School-age is a period of age that ranges from childhood to adolescence. In this life span, oral health related behaviors, beliefs and attitudes are developing and early habits are being established (1-7).

Schoolchildren with poor oral health are more likely to have limited daily activity and missing school days than those with good oral health (2). In fact, the world health organization (WHO) recommends to integrate the oral health promotion and educational programs into the schools’ activities and curriculum (7). Furthermore, oral health promotion should consider other issues such as healthy nutrition, tobacco use, reproductive health, heart disease and obesity (7). It seems however, that the success of the oral health promotion programs among schoolchildren depends heavily on reinforcement at home, especially by mothers (7). A recent study from Iran indicated that targeting oral health promotion programs in school and home settings help to improve children’s oral health status substantially (8). Parents and teachers involvement reinforce promotion programs for children's oral health. Also, with mothers spending relatively more time interacting, fostering and training their children, their behaviors seem to be associated more with children’s oral health (9, 10).

## 2. Objectives

The aim of the present study was to evaluate mothers’ life style behaviors and their association with children oral health.

## 3. Materials and Methods

### 3.1. Participants

The study was a cross sectional survey that was conducted in October and November of 2011. Participants were 11-12 years-old elementary school children and their mothers in Tehran, Iran. This study was approved by the Ethical Committee of Tehran University of Medical Sciences. An informed written consent was taken from all mothers. A cluster sampling design was applied to select a representative sample. Six schools were selected randomly from the list of public primary schools which was provided by the office of Tehran Education Area, Tehran, Iran. In each school two classes of fifth grade students were selected randomly. In total, 383 children together with their mothers were recruited in the study.

### 3.2. Measure

Demographic information such as children’s age and gender, maternal education, age, and occupation, and family income were collected by a questionnaire. All children and their mothers were assessed for body weight and height. Weight was measured to the nearest 0.01 kg and height to the nearest 1 cm. The BMI (Body mass index) values and BMI SD scores were calculated for each participant. The BMI was calculated using the formula weight (in kilograms) divided by the square of height (in meters).

#### 3.2.1. Knowledge

Mothers' oral health knowledge was assessed using 9 items some of which are: gum bleeding is a sign of periodontal disease, fluoride in toothpaste can help to remove stains from teeth, using tooth brush help preventing periodontal diseases, using dental ﬂoss helps preventing periodontal diseases, and dental problems can lead to other health problems. The responses were either yes (1) or no (0). The total knowledge score was obtained by adding up the nine items (ranged 0-9; with higher score indicating higher knowledge).

#### 3.2.2. Attitude

Mothers' attitude toward oral health behavior was assessed using the following three items: tooth brushing, dental flossing, and tongue cleaning on a five-point bipolar scale. The respondents indicated their evaluation on oral health behavior in five dimensions; unhealthy–healthy, negative–positive, annoying–not annoying, useful-not useful, and painful–painless. Internal consistency (α = 0.86) was considered to be acceptable. A summed score for the above three items was considered to be the overall attitude towards oral health behavior (ranged 3 - 15; again with higher score indicating more positive attitude).

#### 3.2.3. Oral Health Behavior

Oral health behavior was assessed using a combined scale Oral Hygiene Behavior (OHB) which was adapted from Buunk-Werkhove and collogues (11). The OHB had been developed by a Delphi method to evaluate actual oral self-care behavior. The OHB covers eight areas including: Frequency of tooth brushing, Moments of tooth brushing, Measure of force of tooth brushing, Duration of tooth brushing, Method of tooth brushing, Fluoride toothpaste, and Using dental floss and tongue cleaner. All items are weighted and the sum OHB score represented oral health behavior and ranged from 0 to 16. Higher score demonstrates higher level of oral self-care behavior (11).

#### 3.2.4. Clinical Examination

Oral hygiene was measured using the Simplified Oral Hygiene Index (OHI-S) of Greene, Vermillion (12). The DMFT (number of decayed, missing and filled teeth) was assessed by a calibrated dentist for children and their mothers. The DMFT examinations were performed in the classrooms under the following conditions: the mother and child seated in a chair, an examiner stood in front of the chair with a headlamp, a mouth mirror and a WHO probe. Then, the periodontal conditions were assessed using Community Periodontal Index (CPI) (13). The CPI index with three scores (0 = healthy gum, 1 = gingival bleeding, 2 = calculus) was applied for evaluation of the periodontal status of the children. The examinations were carried out in accordance with WHO criteria (13) under artificial source of light with a dental mirror and the WHO CPI periodontal probe. These examinations were performed in classrooms by two calibrated dentists.

#### 3.2.5. Calibration

In order to control reliability, the clinical examiners were trained and calibrated by an experienced examiner two weeks prior to the study. The calibration was carried out on a separate group of 14 children. Each child was examined by one of the examiners and re-examined by the second examiner again within 24 hours. Intraclass correlation coefficient (ICC) with a two way random effects model was used to assess intra-examiner reliability in the use of the diagnostic criteria. The examiners were calibrated to 97.4 % and 98.2 % agreement to the referent examiner for OHI-S and CPI respectively, according to the WHO criteria during two weeks prior to the beginning of the field examinations.

### 3.3. Statistical Analysis

To assess content validity of the cognitive measure two indicators were used: the content validity index (CVI) and the content validity ratio (CVR) (14, 15). An expert panel including a biostatistician, health psychologist, health educator, nurse, and dentist evaluated the questionnaire items in terms of relevancy, simplicity and clarity on a Likert-type scale. Furthermore, the essentiality of an item of the questionnaire was rated on a three scale by the panellists. The results of the content validity showed that the CVI was 0.90 and the CVR was 0.88. The ceiling and floor effects were checked for questionnaire and they were in acceptable range for all measures (less than 10 %). Sample size was calculated using G*Power software for multiple logistic regression model, considering a significance level α = 0.01 and power of analysis defined as 1-β = 0.95. Due to cluster sampling approach, sample size was subsequently multiplied by 2 as the design effect of the study. Final sample size required for this study was 360, with an estimate of 60 samples in each school.

We conducted four sets of models in order to identify important children’s or mothers’ characteristics predicting DMFT, CPI and OHB response variables. While the multilevel linear mixed model was most suitable and employed for modeling DMFT and OHB, the proportional odds regression model was used to identify risk indicators of CPI. We applied a two-level random intercept model for each response variable with students as level 1 (n = 383) and schools as level 2 subjects (n = 6) with a random effect for school to take into the account potential intra-cluster correlation. Four different types of multilevel regression models (random intercept models) were fitted for each of the above three response variables (see Tables 2-4). To avoid multicollinearity, standardized forms of continuous variables such as age and BMI of the students and mothers were used in all analyses. The followings are detaile descriptions of the four models:

Model 1: This is a two-level null model of students (level 1) nested within schools (level 2) with only the constant term in the fixed and random parts. Variation in response variable was partitioned across students (within schools) and between schools. The intra-class correlation coefficient indicates the proportion of total variance that may exist among schools at level 2.

Model 2: This is the same as Model 1 with the added variables related to the students’ characteristics (i.e. age, gender, and BMI) and family income in the fixed part of the model. So, this model assesses the effect of the student-related variables and family income on response variables.

Model 3: This is the same as Model 2 with the added variables pertaining to the mothers. Thus, this model assesses the effect of maternal-related characteristics such as age, occupation, educational attainment, and BMI on each of the response variables after adjusting for the student factors and family income.

Model 4: This is the same as Model 3 but with the added variable(s) which is different for each response variable. In Model 4, we added the maternal-related variable of the same type as the response variable. That is, when CPI or DMFT of the students is considered as the response variable, we included CPI or DMFT of the mothers as an additional predictor variable to Model 3. Finally, mothers’ knowledge and attitude were included as the predictor of the student’s OHB index. We considered whether such mothers’ indices introduce exerted effects on the student's indices after adjusting for the student, mother, and family’s other characteristics. In fact, the purpose of this model was to examine whether the influence of mother's oral health is associated with children's oral health.

Missing data were assumed to be missing completely at random and complete cases analysis approach was used for analyses. The likelihood ratio test was employed to compare and contrast the fitness of the models and Wald test applied for testing the variance of random effects. Also, the intraclass correlation was presented for linear models. This basically measures the extent of similarity of the individuals within the same cluster (i.e. school). Data were analyzed with MLwiN 2.1 for multilevel analysis (16).

## 4. Results

Table 1 demonstrates both children and mothers’ demographic characteristics. Average age of the children and their mothers were 11.6 and 38.4 years, respectively. Slightly more children were girls (56.9 %). Half of the children (50 %) had mothers with the education of high school diploma.

Table 2 provides the results of the multilevel models for predictors of the student DMFT. The null model with no predictor (Model 1) revealed a significant variation in student DMFT among schools ((school) = 3.28). However, as indicated before, this result did not take other characteristics of the students into the account. In Model 2, girls had lower DMFT than boys (P = 0.04) but age and BMI of students and family income did not have significant effect on DMFT. Results of model 3 reveals that while students with mothers of higher education have significantly lower DMFT; students with employed mothers have higher count of DMFT. Finally, results of model 4 indicate that DMFT of the students is affected very significantly by mothers' DMFT (P < 0.001).

Table 3 shows the results of the multilevel ordinal logistic models for predictors of the students' gingival health. Model 1 shows a significant variation in students’ CPI among schools ((school) = 0.64). In Model 2, students with higher income families (OR = 0.55, P = 0.042) and female students (OR = 0.46, P < 0.001) were more likely to have lower CPI scores. In Model 3, students with mothers of higher level of education had better gingival health while employment of mothers had negative effect on student's gingival health. Finally, in model 4, students with mothers who have better gingival health also showed to have significantly better gingival health themselves (OR = 1.71, P = 0.035).

Table 4 indicates the results of the multilevel linear regression models for predictors of students' OHB index. The null model with no predictors (Model 1) revealed a significant variation in students’ OHB among schools ((school) = 2.26). In Model 2, girls and students of higher income families had significantly better oral hygiene behavior. In this model as students’ BMI increases the OHB score decreases although the effect of BMI did not reach statistical significance (P = 0.063). In Model 3, students with employed and older mothers had poorer oral hygiene behavior while higher maternal education had significantly positive effect on students' OHB index.

Finally, in Model 4, mothers’ knowledge and attitude about oral health emerged as significant predictors of students’ oral hygiene behavior (P = 0.026 and P = 0.005 respectively). Also, mothers’ higher BMI had a significant negative effect on students’ oral hygiene behavior (P = 0.032). As shown in Tables 2 and 4 there were considerable intraclass correlation for DMFT and OHB index. As expected, the Wald test shows significant variation in random effect at level 2 for each model (P < 0.05). Furthermore, for all response variables, the Likelihood Ratio test indicated significant improvement in likelihood induced just by introduction of an additional variable into the respective model (P < 0.05).

**Table 1.** Demographic and Clinical Features of the Study Sample ^a^

Variables	P value
**Age of students**	11.6 ± 0.2
**Gender**	
Male	165 (43.1)
Female	218 (56.9)
**BMI of student**	19.9 ± 1.1
**DMFT of student**	1.8 ± 0.1
**Family income**	
Low	225 (58.7)
High	158 (41.3)
**Maternal age**	38.4 ± 0.5
**Maternal education**	
Under diploma	108 (28.2)
Diploma	192 (50.1)
University	83 (21.7)
**Maternal occupation**	
Unemployed	242 (63.2)
Employed	141 (36.8)
**Maternal BMI**	25.4 ± 0.4
**Maternal DMFT**	3.8 ± 0.1
**Maternal CPI**	2.1 ± 0.2
**Maternal Knowledge**	3.9 ± 0.9
**Maternal Attitude**	3.6 ± 0.7

^a^ Values are given as No. (%) or Mean ± SD

**Table 2.** Multilevel Linear Regression Models for Predictors of Student DMFT

	Model 1		Model 2		Model 3		Model 4	
	β (SE)	P value	β (SE)	P value	β (SE)	P value	β (SE)	P value
**Fixed effects**								
**Constant**	1.78 (0.47)	< 0.001	1.44 (0.38)	< 0.001	1.21 (0.49)	0.013	1.09 (0.27)	< 0.001
**Age of student**	-	-	0.25 (0.15)	0.108	0.33 (0.27)	0.221	0.28 (0.21)	0.184
**Gender**	-	-	-	-	-	-	-	-
Male	-	-	REF		REF	-	REF	-
Female	-	-	-0.43 (0.21)	0.040	-0.64 (0.25)	0.010	-0.67 (0.26)	0.010
**BMI of student**	-	-	0.11 (0.13)	0.410	0.04 (0.11)	0.729	0.04 (0.11)	0.721
**Family income**	-	-	-	-	-	-	-	-
Low	-	-	REF	-	REF	-	REF	-
High	-	-	-0.17 (0.10)	0.084	-0.22 (0.11)	0.042	-0.24 (0.12)	0.041
**Maternal age**	-	-	-	-	0.18 (0.15)	0.225	0.18 (0.15)	0.23
**Maternal education**	-	-	-	-	-	-	-	-
Under diploma	-	-	-	-	REF	-	REF	-
Diploma	-	-	-	-	-0.45 (0.20)	0.026	-0.53 (0.23)	0.022
University	-	-	-	-	-0.38 (0.18)	0.034	-0.41 (0.20)	0.036
**Maternal occupation**	-	-	-	-	-	-	-	-
Unemployed	-	-	-	-	REF	-	REF	-
Employed	-	-	-	-	0.34 (0.17)	0.047	0.34 (0.17)	0.045
**Maternal BMI**	-	-	-	-	0.13 (0.23)	0.578	0.09 (0.19)	0.631
**Maternal DMFT**	-	-	-	-	-	-	1.82 (0.49)	< 0.001
**Random effects**								
	**Variance (SE)**	**P value**	**Variance (SE)**	**P value**	**Variance (SE)**	**P value**	**Variance (SE)**	**P value**
**Between schools**	3.28 (0.84)	< 0.001	3.39 (0.95)	< 0.001	2.45 (0.73)	< 0.001	2.18 (0.62)	< 0.001
**Intra-class correlation**	0.096	-	0.022	-	0.019	-	0.018	-
-2*Log likelihood	1420.75	-	1210.36	-	983.44	-	865.71	-

**Table 3.** Multilevel Ordinal Logistic Regression Models for Predictors of Student CPI

	Model 1		Model 2		Model 3		Model 4	
	OR	P value	OR	P value	OR	P value	OR	P value
**Fixed effects**								
**Constant**	2.15	-	1.36	-	1.21	-	1.21	-
**Age of student**	-	-	1.07	0.362	1.07	0.352	1.05	0.343
**Gender**	-	-	-	-	-	-	-	-
Male	-	-	REF		REF	-	REF	-
Female	-	-	0.46	< 0.001	0.47	< 0.001	0.52	< 0.001
**BMI of student**	-	-	1.11	0.250	1.06	0.284	1.06	0.292
**Family income**	-	-	-	-	-	-	-	-
Low	-	-	REF		REF	-	REF	-
High	-	-	0.55	0.042	0.54	0.041	0.55	0.038
**Maternal age**	-	-	-	-	1.10	0.365	1.08	0.410
**Maternal education**	-	-	-	-	-	-	-	-
Under diploma	-	-	-	-	REF	-	REF	-
Diploma	-	-	-	-	0.63	0.037	0.61	0.039
University	-	-	-	-	0.48	0.029	0.47	0.030
**Maternal occupation**	-	-	-	-	-	-	-	-
Unemployed	-	-	-	-	REF	-	REF	-
Employed	-	-	-	-	1.36	0.048	1.36	0.048
**Maternal BMI**	-	-	-	-	1.16	0.193	1.8	0.201
**Maternal CPI**	-	-	-	-	-	-	1.71	0.035
**Random effects**								
	**Variance (SE)**	**P value**	**Variance (SE)**	**P value**	**Variance (SE)**	**P value**	**Variance (SE)**	**P value**
**Between schools**	0.64 (0.19)	< 0.001	0.38 (0.11)	< 0.001	0.22 (0.06)	< 0.001	0.21 (0.06)	< 0.001
-2*Log likelihood	856.23		526.41		431.15	-	410.19	-

**Table 4.** Multilevel Linear Regression Models for Predictors of Student OHB Index

	Model 1		Model 2		Model 3		Model 4	
	β (SE)	P value	β (SE)	P value	β (SE)	P value	β (SE)	P value
**Fixed effects**								
**Constant**	11.15 (3.25)	< 0.001	10.28 (2.76)	< 0.001	8.95 (2.67)	< 0.001	13.47 (4.68)	0.004
**Age of student**	-	-	0.61 (0.83)	0.463	0.58 (0.94)	0.536	0.54 (0.93)	0.561
**Gender**	-	-	-	-	-	-	-	-
Male	-	-	REF	-	REF	-	REF	-
Female	-	-	4.36 (2.02)	0.031	6.74 (2.68)	0.012	5.51 (2.19)	0.012
**BMI of student**	--	-	-0.56 (0.30)	0.063	-0.72 (0.36)	0.046	-0.82 (0.40)	0.041
**Family income**	-	-	-	-	-	-	-	-
Low	-	-	REF	-	REF	-	REF	-
High	-	-	1.23 (0.58)	0.033	1.08 (0.52)	0.037	1.08 (0.51)	0.033
**Maternal age**	-	-	-	-	-0.75 (0.34)	0.028	-0.66 (0.31)	0.033
**Maternal education**	-	-	-	-	-	-	-	-
Under diploma	-	-	-	-	REF	-	REF	-
Diploma	-	-	-	-	1.94 (0.82)	0.018	1.83 (0.72)	0.011
University	-	-	-	-	3.22 (1.12)	0.004	5.11 (1.72)	0.003
**Maternal occupation**	-	-	-	-	-	-	-	-
Unemployed	-	-	-	-	REF	-	REF	-
Employed	-	-	-	-	-0.84 (0.39)	0.031	-0.81 (0.37)	0.028
**Maternal BMI**	-	-	-	-	-1.71 (0.81)	0.035	-1.66 (0.77)	0.032
**Maternal Knowledge**	-	-	-	-	-	-	1.32 (0.59)	0.026
**Maternal Attitude**	-	-	-	-	-	-	1.84 (0.66)	0.005
**Random effects**								
	**Variance (SE)**	**P value**	**Variance (SE)**	**P value**	**Variance (SE)**	**P value**	**Variance (SE)**	**P value**
**Between schools**	2.26 (1.11)	0.042	1.18 (0.56)	0.035	1.15 (0.52)	0.026	1.12 (0.47)	0.018
**Intra-class correlation**	0.121	-	0.071	-	0.068	-	0.052	-
-2*Log likelihood	2537.15	-	1289.42	-	785.36	-	560.34	-

## 5. Discussion

Children’s health behavior is influenced by their parents’ knowledge and beliefs, which affect oral hygiene and healthy eating habits. To our knowledge, no study is available to assess the influence of the mothers’ oral health on their children in Iran. In general, this study demonstrated that mothers’ BMI, knowledge, attitude, and oral health behavior indices were associated with children's oral health. Also in this study, we found that female gender, higher family income, higher mother's education, and lower mother's DMFT, were all associated with lower children's DMFT. Gender plays an important role in oral health behavior. Girls seem more likely to have positive attitude toward oral health behavior and higher intention to perform behaviors (such as tooth brushing and flossing) than boys. A possible explanation of this could be aesthetic issues. Girls care for their oral health more than boys do. This fact could be influenced by parents, especially mothers (17). In Iran, mothers are more sensitive to their girls in their aesthetic issues. Therefore, this could affect girls' oral health and DMFT. Similar results were found in previous studies (5, 18, 19). In our study, low family income was associated significantly with the childrens’ dental caries, DMFT, and gingival health (i.e. CPI).

A higher prevalence of dental caries among families with low income has also been reported previously in some studies (20-22). All in all, studies indicated that socioeconomic status is an important predictor of dental caries (22-24). Low income may also be associated with lower education level, impaired understanding of the value of health and poor access to oral health care facilities. Another study from Iran revealed dental insurance coverage as a predisposing factor for dental attendance and also check-ups among adults in Tehran (25). Furthermore, living in a family with low income puts the children at risk of not using tooth brush and dental floss (26). The present study indicated that mothers’ education was one of the variables influencing DMFT, CPI, as well as, health behavior in children. These results were consistent with the family income.

Socioeconomic status is measured by family income and the level of parental education (27). Families in high level of education probably have higher level of knowledge on importance of oral health care, positive attitude toward oral health behavior, support their children's tooth brushing efforts, eating healthy food and select fewer sweets (19, 28, 29). Parents play a central role transferring health-related information, and supporting healthy behavior of their children (30). Furthermore, mothers are considered as role models in health behavior of their children. They transfer values, norms, and accepted behaviors to their children (31). Therefore, parents especially mothers serve as social models for their children (32). The results revealed that mothers' DMFT and CPI had strong influence on children's DMFT and CPI. These findings are partly important because most oral health education are individual based and blame individuals for failure and getting oral disease. Hence, interventions often focus only children in schools. This study expands our knowledge emphasizing the role of the family on children's caries. Similar results were reported in previous studies (33, 34). This study has several limitations. First, the mother and children who participated in the study were not completely representative of Iranian mothers and children. Furthermore, this study was a cross sectional study rather than a longitudental study. Therefore, additional studies should be performed in a more representative sample of Iranian mothers and their children to assess the oral health change in a longitudinal design.

In conclusion, mothers’ knowledge, educational status, attitude, oral health behaviors, DMFT and CPI were all significantly associated with their children oral health status. Educational interventions which focus on both children and their mothers will obtain more promising results.
